# Effectiveness of a Multidisciplinary Team-Approach on Central-Line–Associated Bloodstream Infections

**DOI:** 10.1017/ash.2021.13

**Published:** 2021-07-29

**Authors:** Geehan Suleyman, Melissa Ahrens, Ann Keegan

## Abstract

**Background:** Although there has been a significant reduction in central-line–associated bloodstream infection (CLABSI) rates in the past decade with the implementation of evidence-based practices, an estimated 30,100 CLABSI occur each year in acute-care facilities. CLABSIs are associated with increased length of stay, cost, morbidity, and mortality, and they are preventable. In this study, we assessed the impact of a multidisciplinary team approach on CLABSI rates at a 319-bed teaching hospital in northwestern Ohio. **Methods:** In this before-and-after retrospective study, we compared the CLABSI rate per 1,000 central-line days, standardized infection ratio (SIR), and standardized utilization ratio (SUR) in the preintervention period (January 1, 2016, to December 31, 2018) to those of the intervention period (January 1, 2019, to December 31, 2020). Despite hospital-wide nursing education focusing on central-line maintenance in 2017, our SIR and SUR remained above the national benchmark. Starting in August 2018, we began to focus on insertion practices and physician education. An infection preventionist observed resident central-line insertion training and noted that there was no emphasis on infection prevention measures. There was a best practice knowledge gap. Thus, the indications for central-line use were updated, the insertion checklist was standardized, and the vascular access policy was revised to limit femoral and internal jugular vein use. Infection prevention training was provided to all providers involved in central-line insertions. Nurses were tasked with observing insertion of every central line and stopping the procedure if there is was an observed break in sterile technique. A central-line report listing indications and duration was developed and was sent to the nursing directors who assessed daily need with providers and prompted removal of unnecessary lines. The infection prevention medical director provided CLABSI prevention education to providers. **Results:** The CLABSI rate per 1,000 central-line days decreased from 0.90 in the preintervention period to 0.34 in the postintervention period, resulting in a 62% reduction in CLABSI rate. The SIR decreased from 0.886 to 0.323 (p-value <0.05), yielding a 64% reduction. The SUR also decreased from 1.156 to 0.874 (p-value <0.001) with a 24% reduction. **Conclusion:** A multidisciplinary team-approach with emphasis on standardized insertion checklist to ensure adherence to sterile technique and prompt removal of unnecessary central lines, and physician insertion training focusing on IP practices may potentially reduce CLABSI rates.

**Funding:** No

**Disclosures:** None

